# Reply to “Conformational fitting of a flexible oligomeric substrate does not explain the enzymatic PET degradation”

**DOI:** 10.1038/s41467-019-13493-8

**Published:** 2019-12-06

**Authors:** Hogyun Seo, In Jin Cho, Seongjoon Joo, Hyeoncheol Francis Son, Hye-Young Sagong, So Young Choi, Sang Yup Lee, Kyung-Jin Kim

**Affiliations:** 1 School of Life Sciences (KNU Creative BioResearch Group), KNU Institute for Microorganisms, Kyungpook National University, Daehak-ro 80, Buk-gu, Daegu, 41566 Republic of Korea; 2 Metabolic and Biomolecular Engineering National Research Laboratory, Systems Metabolic Engineering and Systems Healthcare Cross Generation Collaborative Laboratory, Department of Chemical and Biomolecular Engineering (BK21 Plus Program), Institute for the BioCentury, Korea Advanced Institute of Science and Technology (KAIST), 291 Daehak-ro, Yuseong-gu, Daejeon, 34141 Republic of Korea; 3 BioProcess Engineering Research Center and BioInformatics Research Center, KAIST, 291 Daehak-ro, Yuseong-gu, Daejeon, 34141 Republic of Korea

**Keywords:** Biochemistry, Biotechnology

**Replying to** Wei et al. *Nature Communications* 10.1038/s41467-019-13492-9 (2019)

The manuscript entitled “Conformational fitting of a flexible oligomeric substrate does not explain the enzymatic PET degradation” by Wei et al.^1^ raises a question on the conclusion reached in our published work, particularly regarding the docking calculations of PET substrate into the PETase from *Ideonella sakaiensis* (*Is*PETase)^2^. The authors showed the ethylene glycol torsion angle *Ψ* in an amorphous PET material of 0.25 mm thickness (Goodfellow Cambridge Ltd.) using solid-state nuclear magnetic resonance experiments and determined *trans*/*gauche* ratio of 9:91 at 30 °C in good agreement with the previous report on amorphous PET (14 ± 5%)^3^. Based on the result, they suggested that the conformation of the docked 2-HE(MHET)_4_ in our published work that showed a *trans* content of ethylene glycol higher than 25% is rarely present in amorphous PET polymer chains, and claimed that the residues in subsite IIb and IIc we suggested are unlikely to interact with the two MHET moieties of 2-HE(MHET)_4_. To support the latter statement, they further demonstrated that the transition from *gauche* to *trans* conformation is highly restricted in amorphous PET at 30 °C by magic-angle-spinning nuclear magnetic resonance method. They also suggested that, instead of the perfect accommodation, the key factor facilitating the substrate binding seems to be the fragile contact between the phenylene moieties and the surrounding hydrophobic residues. In general, we agree that the comments by Wei et al. provide concrete experimental results showing that ethylene glycol units of amorphous PET polymer do not have the free rotational properties to fit into its substrate binding site as the form of 2-HE(MHET)_4_ at 30 °C. Thus, the study provides the scientific community with useful information on PET degradation by *Is*PETase.

However, our previous docking calculations^2^ were independent of temperature setting and not restricted to the temperature of 30 °C. Although *Is*PETase cannot maintain its activity at high temperature due to its low thermal stability, efforts to increase thermal stability of the enzyme have already been reported^4^. Thus, an engineered *Is*PETase with high thermal stability might be able to accommodate the PET substrate in the manner we presented at temperatures higher than 30 °C as the *trans* content of the material was increased to 56% at 70 °C^1^.

Moreover, we did not use the *Goodfellow* amorphous PET in our published works^2,4^, which counters the comment that *Is*PETase showed almost “no” activity against crystalline PET polymer^1^. It was also previously shown that the semi-crystalline PET material exhibits much higher *trans* conformation of ethylene glycol than amorphous one at ambient temperature^3^. Thus, we believe that the authors’ claims regarding the residues in subsite IIb and IIc may not apply under all conditions. In sum, while we agree that Wei et al. provide useful evidences that the used docking calculation is not suitable in amorphous PET at low temperature such as 30 °C, their observations are not necessarily incompatible with the general findings of our previous work^2^. We anticipate that Wei et al.’s work and our own will inspire future studies aimed at unraveling the exact mechanisms of PET degradation.

## Data Availability

Data supporting the findings of this study are available within the article and from the corresponding author upon reasonable request.

## References

[1] Wei, R. et al. Conformational fitting of a flexible oligomeric substrate does not explain the enzymatic PET degradation. *Nat*. *Commun*. (2019). 10.1038/s41467-019-13492-9 PMC6897938 31811142

[2] Joo S (2018). Structural insight into molecular mechanism of poly(ethylene terephthalate) degradation. Nat. Commun..

[3] Schmidt-Rohr K, Hu W, Zumbulyadis N (1998). Elucidation of the chain conformation in a glassy polyester, PET, by two-dimensional NMR. Science.

[4] Son HF (2019). Rational protein engineering of thermo-stable PETase from *Ideonella sakaiensis* for highly efficient PET degradation. ACS Catal..

